# Orbitally forced and internal changes in West African rainfall interannual-to-decadal variability for the last 6000 years

**DOI:** 10.1007/s00382-023-07023-y

**Published:** 2023-11-30

**Authors:** Julien Crétat, Sandy P. Harrison, Pascale Braconnot, Roberta d’Agostino, Johann Jungclaus, Gerrit Lohmann, Xiaoxu Shi, Olivier Marti

**Affiliations:** 1 Science Partners, Paris, France; 2 Centre de Recherches de Climatologie, UMR 6282 Biogéosciences, CNRS Université de, Bourgogne, France; 3 Department of Geography and Environmental Science, University of Reading, Reading, RG6 6AB UK; 4 Laboratoire des Sciences du Climat et de l’Environnement-IPSL, Unité Mixte CEA-CNRS-UVSQ, Université Paris-Saclay, Orme des Merisiers, Gif-sur-Yvette, France; 5 Max Planck Institute for Meteorology, Hamburg, Germany; 6 Institute of Atmospheric Sciences and Climate (ISAC), National Research Council of Italy, Bologna, Italy; 7 Alfred Wegener Institute, Helmholtz Centre for Polar and Marine Research, Bremerhaven, Germany; 8 Department of Environmental Physics and MARUM, University of Bremen, Bremen, Germany

**Keywords:** West African monsoon, Multi-centennial rainfall variability, Interannual-to-decadal rainfall variability, Ocean–atmosphere climate modes, Transient climate model simulations, Holocene

## Abstract

Recent variability in West African monsoon rainfall (WAMR) has been shown to be influenced by multiple ocean–atmosphere modes, including the El Niño Southern Oscillation, Atlantic Multidecadal Oscillation and the Interdecadal Pacific Oscillation. How these modes will change in response to long term forcing is less well understood. Here we use four transient simulations driven by changes in orbital forcing and greenhouse gas concentrations over the past 6000 years to examine the relationship between West African monsoon rainfall multiscale variability and changes in the modes associated with this variability. All four models show a near linear decline in monsoon rainfall over the past 6000 years in response to the gradual weakening of the interhemispheric gradient in sea surface temperatures. The only indices that show a long-term trend are those associated with the strengthening of the El Niño Southern Oscillation from the mid-Holocene onwards. At the interannual-to-decadal timescale, WAMR variability is largely influenced by Pacific–Atlantic – Mediterranean Sea teleconnections in all simulations; the exact configurations are model sensitive. The WAMR interannual-to-decadal variability depicts marked multi-centennial oscillations, with La Niña/negative Pacific Decadal Oscillation and a weakening and/or poleward shift of subtropical high-pressure systems over the Atlantic favoring wet WAMR anomalies. The WAMR interannual-to-decadal variability also depicts an overall decreasing trend throughout the Holocene that is consistent among the simulations. This decreasing trend relates to changes in the North Atlantic and Gulf of Guinea Sea Surface Temperature variability.

**Supplementary Information:**

The online version contains supplementary material available at 10.1007/s00382-023-07023-y.

## Introduction

West African society is highly dependent on rainfall because it is reliant on rain-fed agriculture under semi-arid conditions. Up to 55% of the annual rainfall occurs during the northern-hemisphere summer monsoon season (Wang and Ding 2008), between July and September (JAS), when low-level south-westerly winds from the Atlantic bring large amounts of moisture into West Africa due to land-sea thermal contrast. However, there is considerable variation in West African Monsoon Rainfall (WAMR) on multiple timescales, making West Africa one of the most vulnerable regions of the world.

Most studies of the drivers of WAMR variability have focused on the recent past to understand the causes of the 1970s and 1980s droughts and the recent partial recovery. They have shown that multiple mechanisms are responsible for the marked fluctuations in WAMR on interannual and decadal timescales. On the interannual timescale, wet WAMR anomalies are favored during the negative phase of the El Niño Southern Oscillation (ENSO) and by cold sea surface temperature (SST) anomalies in the equatorial Atlantic (e.g., Janicot et al. 2001; Joly et al. 2007; Joly and Voldoire 2009; Losada et al. 2010; Rodriguez-Fonseca et al. 2011; Mohino et al. 2011a; Rodriguez-Fonseca et al. 2015). Pulsations of the Saharan heat low have also been shown to favor moisture transport from the Atlantic and Mediterranean Sea into West Africa (Lavaysse et al. 2016), with e.g. warm Mediterranean SST anomalies accompanied by wet WAMR anomalies (Rowell 2003; Fontaine et al. 2010; Gaetani et al. 2010).

On the decadal-to-multidecadal timescale, WAMR is influenced by the Atlantic Multidecadal Oscillation (AMO: Knight et al. 2006; Zhang and Delworth 2006; Dima and Lohmann 2007; Ting et al. 2009; Mohino et al. 2011b; Martin et al. 2014; Martin and Thorncroft 2014; Monerie et al. 2019), and the Interdecadal Pacific Oscillation (IPO, Villamayor and Mohino 2015; Dong and Dai 2015). Wet WAMR anomalies are favored in the positive phase of the AMO (AMO +) and in the negative phase of the IPO (IPO-). The AMO + is characterized by warm SST anomalies in the North Atlantic that increase the interhemispheric gradient, favoring moisture advection from the Atlantic into West Africa. The IPO- is characterized by cold SST anomalies in the tropics, most prominently in the Pacific, and in the northern and southern parts of the eastern Pacific, and warm SST anomalies in the western Pacific, poleward of ~ 25°. The IPO- promotes wet WAMR anomalies through the atmospheric bridge (Walker circulation) connecting downward movement over the central Pacific in response to local cold SST anomalies with uplift over West Africa (Villamayor and Mohino 2015). In contrast, dry WAMR anomalies occur in the negative phase of the AMO (AMO-) and the positive phase of the IPO (IPO +). The effects of the AMO and IPO can also be combined (Joshi et al. 2022), with much more WAMR variance explained when the two modes co-occur: AMO + and IPO- co-occurrence leads to much larger wet WAMR anomalies than those associated with AMO + and IPO- individually. The SST interhemispheric dipole also strongly regulates the northern hemisphere summer monsoon, including WAMR, on the decadal-to-multidecadal timescale, with monsoon strengthening (weakening) as the dipole increases (decreases) through planetary-scale adjustments of the atmospheric circulation (Xue et al. 2022).

The extent to which WAMR variability and its driving ocean–atmosphere modes respond to changes in external forcing is not well known. This is partly because there are relatively few paleoclimate records of rainfall variability over West Africa and partly because, until recently, climate model simulations have focused on short periods of time such as the end of the African Humid period (Claussen et al. 1999; Renssen et al. 2003; Liu et al. 2006, 2007; Collins et al. 2017), the last millennium (Stevenson et al. 2018; Zhang et al. 2021) or the response to historic and future changes in CO_2_ (Monerie et al. 2012, 2020; Roehrig et al. 2013). However, several modelling groups have now run fully coupled transient simulations for the past 6,000 years (see e.g. analyses in Carré et al. 2021; Parker et al. 2021; Dallmeyer et al. 2021; Shi et al. 2022) and these simulations provide an opportunity to examine the relationship between WAMR multiscale variability and changes in the modes associated with this variability.

Both paleoclimate records (Hernández et al. 2020) and climate simulations (Braconnot et al. 2019b) suggest that all of the modes of variability affecting the WAMR were active during the Holocene. The main difference between the modern climate and that of the mid- to late Holocene was the weakening of the interhemispheric thermal gradient during boreal summer from mid- to late Holocene, and the strengthening of this gradient during austral summer, induced by orbital forcing (obliquity and precession). This resulted in a southward shift of the Inter-Tropical Convergence Zone (ITCZ) over the ocean and in a weakening of land-sea contrast during boreal summer, and hence increased aridity over West Africa from the mid- to late-Holocene. Analysis of two transient climate simulations of the last 6,000 years (Braconnot et al. 2019b) showed (1) that both WAMR and Indian summer monsoon rainfall (ISMR) decreased gradually from the mid- to late Holocene at the millennial timescale, but with multi-centennial fluctuations internally generated by inter-basin teleconnections; (2) these internally generated fluctuations counteract or exacerbate the orbitally-forced drying trend, leading to strong compound events such as multi-centennial droughts, and (3) that interannual variability responded to orbital forcing through changes in the mean climate state and the response differs regionally: ISMR interannual variability increased from the mid- to late Holocene while WAMR variability decreased. Changes in variability have also been reported in paleoenvironmental records for e.g. ENSO (Carré et al. 2014; Cobb et al. 2013; Emile‐Geay et al. 2016) and the North Atlantic Oscillation (NAO; Rimbu et al. 2003; Olsen et al. 2012).

This study focuses on regional-scale changes in WAMR mean state and variability. The aims are twofold. Firstly, we examine whether the relationships between the WAMR and SST co-variability at different timescales established by Braconnot et al. (2019b) are model-dependent by analysing two additional transient simulations. Secondly, we assess how scale interactions may have affected WAMR variance during the Holocene. Section 2 presents the transient simulations and the method used to examine WAMR-SST co-variability. Sections3 and 4 address the WAMR-SST relationship and changes in the interannual-to-decadal relationship, respectively. Section 5 discusses the results and summarises the main conclusions.

## Data and methods

### Transient simulations

We analyzed four transient simulations of the past 6,000 years run with the AWI, MPI and IPSL-Vlr01 and IPSL-Sr02 Earth System Models (Table 1; Supplementary Information). The IPSL-Sr02 model has a higher resolution in the atmosphere than IPSL-Vlr01 (Table 1) and more sophisticated physics, including an 11-layer hydrological model and a prognostic 3-layer snow model. This simulation also includes dynamic vegetation whereas the IPSL-Vlr01 simulation was made using prescribed vegetation for 1850 CE (Dufresne et al. 2013). The MPI and AWI simulations also include dynamic vegetation (Table 1). Although these models differ in several respects, they both use the ECHAM atmosphere model and the JSBACH land-surface model (see Supplementary Information). The four simulations were forced by annual changes in orbital parameters and greenhouse gases (GHGs). The orbital parameters were calculated following Berger (1978). GHGs in the IPSL-Vlr01 and IPSL-Sr02 were updated annually based on ice core measurements following Otto‐Bliesner et al. (2017) while the AWI simulation was forced using ice core measurements from Köhler et al. (2017) and the MPI simulation used updated measurements as specified in Dallmeyer et al. (2021). However, the differences between the GHG forcings are small and not sufficient to cause differences in the simulated trajectories between the four simulations.

**Table 1:** Main characteristics of the four transient simulations used in this study. (See Supplementary for fuller descriptions)

Model	Simulation name	Horizontal resolution		Vegetation	Reference
		Atmosphere	Ocean		
AWI-ESM2	AWI	T63	Variable (ca 15 km in polar and coastal regions; up to 135 km elsewhere	Dynamic	Lamping et al. (2021); Shi et al. (2022)
MPI-ESM	MPI	T63	1.5°	Dynamic	Bader et al. (2020); Dallmeyer et al. (2021)
IPSL-TR5AS	IPSL-Vlr01	3.75° × 1.875°	2°	Prescribed	Braconnot et al. (2019a, 2019b)
IPSL-TR6AV	IPSL-Sr02	2.5° × 1.25°	2°	Dynamic	

### Assessing WAMR–SST co-variability

Wavelet analyses of the WAMR anomalies for the last 6000 years show that the four simulations all exhibit variability on (a) interannual-to-decadal and (b) millennial timescales. Following Braconnot et al. (2019b), we applied an empirical orthogonal function (EOF) analysis to June‐to‐September seasonal WAMR and SST indices (Table 2) over the last 6000 years filtered on two distinct bands (Fast Fourier Transform filtering): 2-20-year to reflect WAMR and SST interannual-to-decadal variability and > 500-year to focus on orbitally-forced trends of WAMR and SST mean state. One EOF analysis was performed for each band and each simulation, providing 8 EOFs (4 simulations × 2 bands) in total. We have performed the same analysis for the 50-500-year band-pass filtered window but there was no significant trend and we therefore do not use this window for further analysis.

**Table 2:** List of area-averaged indices used to study the co-variability between West African rainfall and the main ocean–atmosphere modes of variability for the last 6,000 years

Indices	Acronym	Location
West African rainfall	WAMR	7–18°N; 15°W to 20°E (land points)
Atlantic multidecadal oscillation	AMO	difference between North Atlantic SST (equator to 60°N; 80°W to 0) and global SST in the 60°S to 60°N band
Mediterranean	MED	30–50°N; 8°W to 45°E
North tropical Atlantic	NTA	5°S to 25°N; 55–15°W
South tropical Atlantic	STA	20°S to equator; 30°W to 10°E
Atlantic dipole	TAD	NTA minus STA
Tropical Atlantic	ATL3	3°S to 3°N; 20°W to 0
West Indian Ocean	WIO	10°S to 10°N; 50–70°E
East Indian Ocean	EIO	10°S to equator; 90–110°E
Indian Ocean dipole	IOD	WIO minus EIO
Niño 3.4 region	NINO34	5°S to 5°N; 170–120°W

The EOF analyses were used as a mathematical framework (i) to summarize the main WAMR–SST cross-correlation patterns at the millennial (500-year low-pass filter) and interannual-to-decadal (2-20-year band-pass filter) timescales, and (ii) to isolate the main modes contributing to decreased WAMR interannual-to-decadal variability from the mid- to late Holocene based on explained variance.

## Multiscale relationship between WAMR and SST

### Temporal changes in mean state and interannual-to-decadal variability

**Fig. 1:**
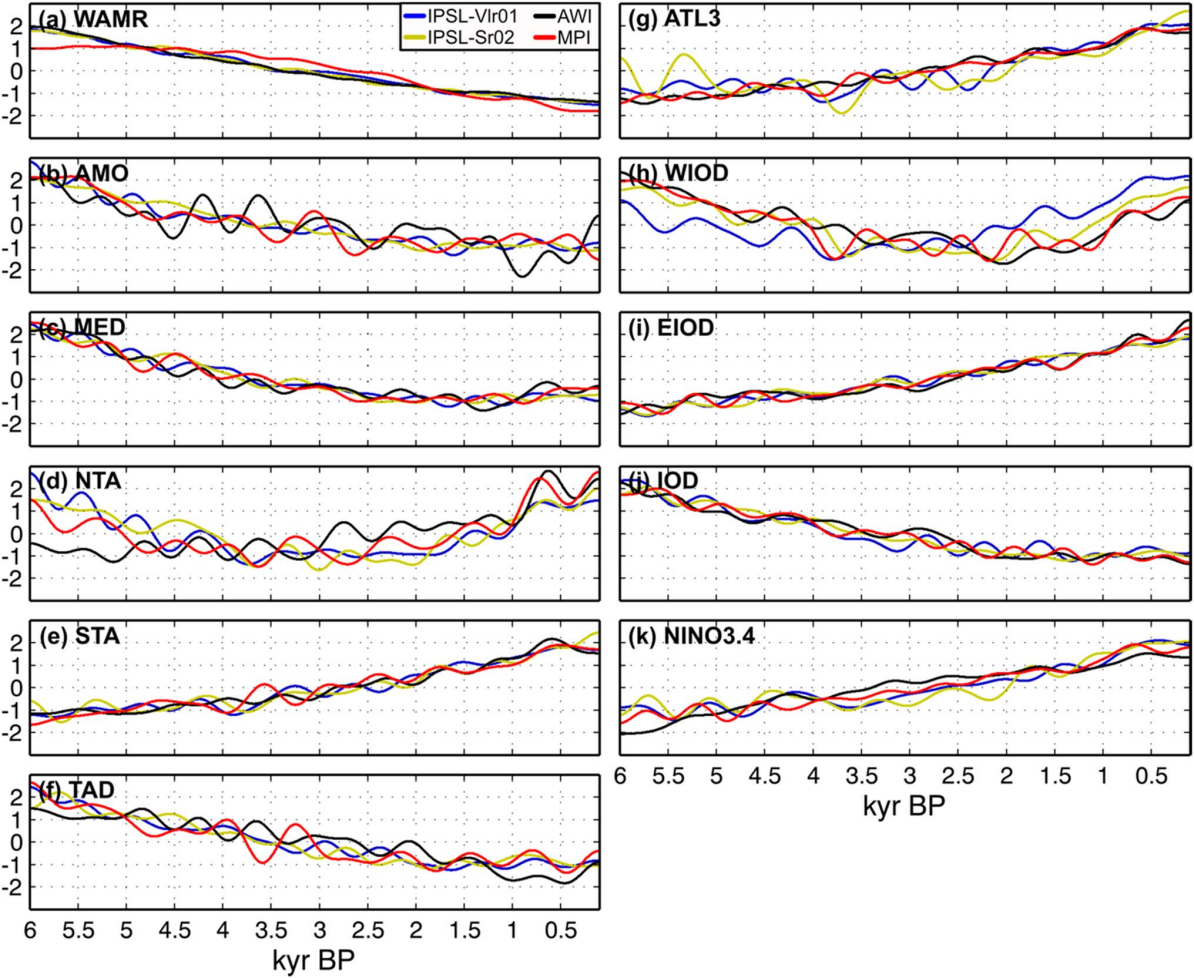
Mid- to late Holocene long-term changes induced by orbital and trace gas forcing for (**a**) WAMR and (b-k) SST indices. Long-term changes are assessed through 500-year low-pass Fast Fourier Transform filtering of standardized WAMR and SST indices (mean = 0 and standard deviation = 1). Each curve represents one transient simulation. The abbreviations in each panel stands for West African rainfall (WAMR), Atlantic Multidecadal Oscillation (AMO), Mediterranean Sea (MED), North and South tropical Atlantic (NTA and STA), Atlantic dipole (TAD), tropical Atlantic (ATL3), western and eastern parts of the Indian Ocean dipole (WIOD and EIOD), Indian Ocean dipole (IOD) and Niño 3.4 region (NINO3.4). The spatial domains considered for WAMR and SST indices are detailed in Table 2

The absolute amount of WAMR precipitation varies between the four models and there are also differences in the absolute values of the simulated SSTs in each of the examined regions (Fig. S1a). However, all of the models show similar and consistent trends in the long-term mean state changes as diagnosed using the 500-year low-pass filtered WAMR and SST indices (Fig. 1). The mid- to late Holocene transition is marked by (i) SST cooling in the AMO, MED, TAD and IOD regions, (ii) SST warming in the STA, EIOD and NINO3.4 regions and (iii) a near-linear decline in WAMR (Fig. 1). Importantly, the WAMR decline remains nearly linear when considering the unfiltered index (Fig. S2), suggesting multi-centennial droughts are not strong nor widespread enough to lead to abrupt drying of the West African monsoon at the regional scale (i.e., 7–18°N by 15°W–20°E) in the four transient simulations. These trends reflect the weakening of both interhemispheric and land-sea contrast during boreal summer from the mid-Holocene onwards, consistent with the known effect of orbital forcing on climate during the Holocene (Joussaume et al. 1999; Liu et al. 2004; Zhao et al. 2005; Zhao and Harrison, 2012). However, all of the simulations show complex SST trends in the NTA and WIOD regions (Fig. 1d and h): SSTs cool in both regions up to ~ 3 kyr BP and warm subsequently. Furthermore, these are the two regions in which one model exhibits a slightly different evolution than others: AWI in the case of NTA and IPSL-Vlr01 for WIOD. Such complex trends probably result from interactions between the effects of orbital and greenhouse gas forcing. The near-linear trend in the tropical Atlantic dipole (Fig. 1f) indicates that the long-term changes are driven more by changes in the southern than the northern tropical Atlantic. Similarly, the long-term changes in the Indian Ocean dipole (Fig. 1j) are constrained more by changes in the eastern than the western Indian Ocean (Falasca et al. 2022).

**Fig. 2:**
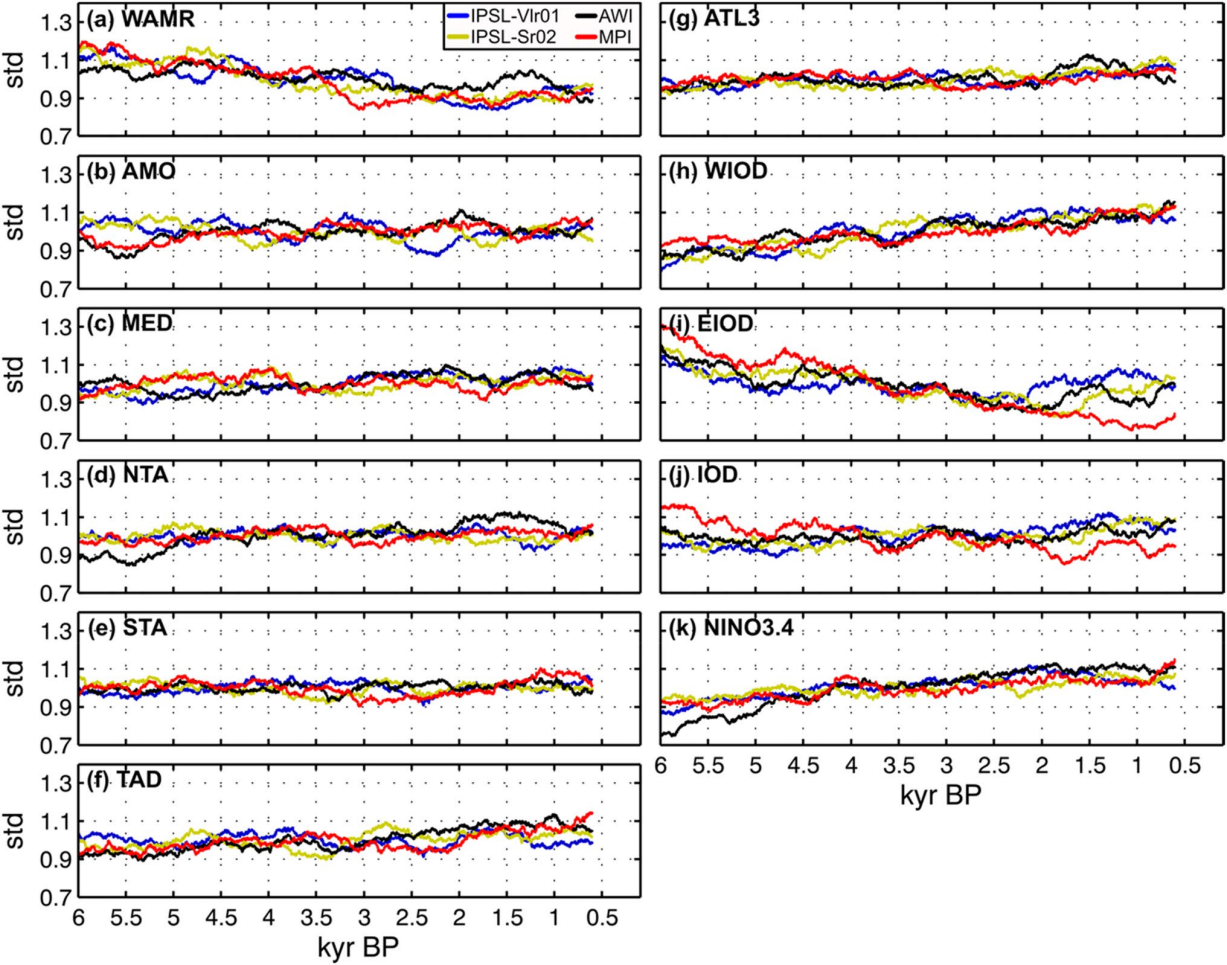
Mid- to late Holocene changes in interannual-to-decadal variability of (**a**) WAMR and (**b**-k) SST indices. Interannual-to-decadal timescale is isolated through a 2–20-year band-pass Fast Fourier Transform filtering of standardized WAMR and SST indices (mean = 0 and standard deviation = 1). Interannual-to-decadal variability is defined as the standard deviation of the 2–20-year filtered timeseries computed along 500-year sliding windows with one year increment between the windows. Each curve represents one transient simulation. The abbreviations and spatial domains considered for WAMR and SST indices are detailed in Table 2

The interannual-to-decadal variability in the WAMR and SST indices also varies between the four models (Fig. S1b), but again there are similarities in the trends in variability (defined as the moving standard deviation of 2–20-year band-pass filtered WAMR and SST indices). WAMR interannual-to-decadal variability decreases from the mid- to late Holocene (Fig. 2a), especially between 6 to ~ 2 kyr BP, in line with the long-term weakening of WAMR (Fig. 1a). This trend is marked by multi-centennial oscillations (Fig. 2a). Both the long-term trend in WAMR interannual-to-decadal variability and the existence of multi-centennial oscillations superimposed on this trend are robust across the simulations, although there are differences in the magnitude of the trend and in the amplitude and timing of multi-centennial oscillations between the models.

Only three of the SST indices show a long-term trend in interannual-to-decadal variability: the WIOD and NINO3.4 with increased variability over the last 6,000 years (Fig. 2h, k) and the EIOD region with decreased variability from 6 to ~ 3 kyr BP (Fig. 2i). These mid- to late Holocene trends are consistent with warming and increased variability of the eastern equatorial Pacific SST (Figs. 1k and 2k), the stronger imprint of ENSO on western tropical IO, and the weakening of upwelling in the eastern equatorial IO reported in previous studies (Falasca et al. 2022). The shape of the trends in the mean state and variability are similar for NINO3.4 (Figs. 1k and 2k) and broadly similar for EIOD (Figs. 1i and 2i), IOD (Figs. 1j and 2j), except for MPI which behaves somewhat differently. These similarities suggest a link between mean state and variability in the tropical Indo-Pacific belt. However, the trends in the mean state and the variability in the WIOD region are not similar: the SST mean state has a non-linear trend reflecting the mixed influence of orbital and trace gas forcing (Fig. 1h) while interannual-to-decadal SST variability has a near linear trend (Fig. 2h) similar to that associated with NINO3.4 SST (Fig. 2k). None of the other SST indices show long-term trends in interannual-to-decadal variability (Fig. 2), despite long-term trends in their mean state (Fig. 1). In addition, all SST indices display multi-centennial oscillations that are not necessarily in phase across the regions and the simulations. Some multi-centennial oscillations in the AMO and tropical Atlantic sectors (Fig. 2b, d, e, g), however, show similarities with multi-centennial oscillations of WAMR interannual-to-decadal variability, indicating that Atlantic internal modes of variability were important drivers of WAMR throughout the Holocene regardless of the climate mean state.

Despite absolute differences between the models, the mid- to late Holocene changes in both the mean state and interannual-to-decadal variability of WAMR and regional SSTs are very consistent among the models. There is, however, no straightforward link between mean state changes and interannual-to-decadal variability changes for each of the indicators. The reduction in WAMR mean state from the mid- to late Holocene is near linear and results from orbitally-driven temperature changes. The decreasing interannual-to-decadal variability of the WAMR appears to be linked to changes in tropical Indo-Pacific and, to a lesser extent, tropical Atlantic SST variability, with multi-centennial oscillations phased with the AMO superimposed.

### Changes in cross-correlation patterns

The EOF framework was applied to both 500 year low-pass and 2–20 year band-pass filtered WAMR and SST indices to summarize the main teleconnection patterns between WAMR and SST for the mean state and interannual-to-decadal variability, respectively.

#### Mean state

**Fig. 3:**
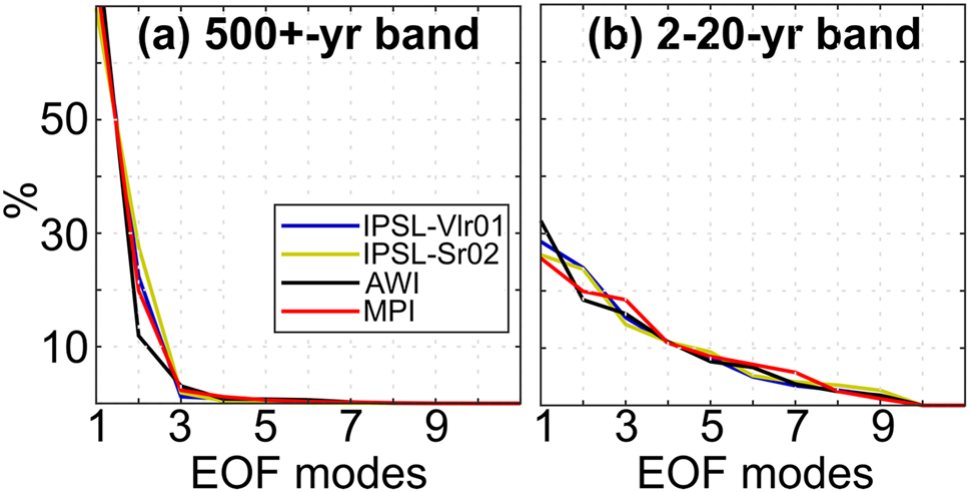
Variance explained by the first 10 EOF modes of the WAMR – SST correlation matrix filtered in the (**a**) 500‐year low‐pass and (**b**) 2–20-year bands for each transient simulation

**Fig. 4:**
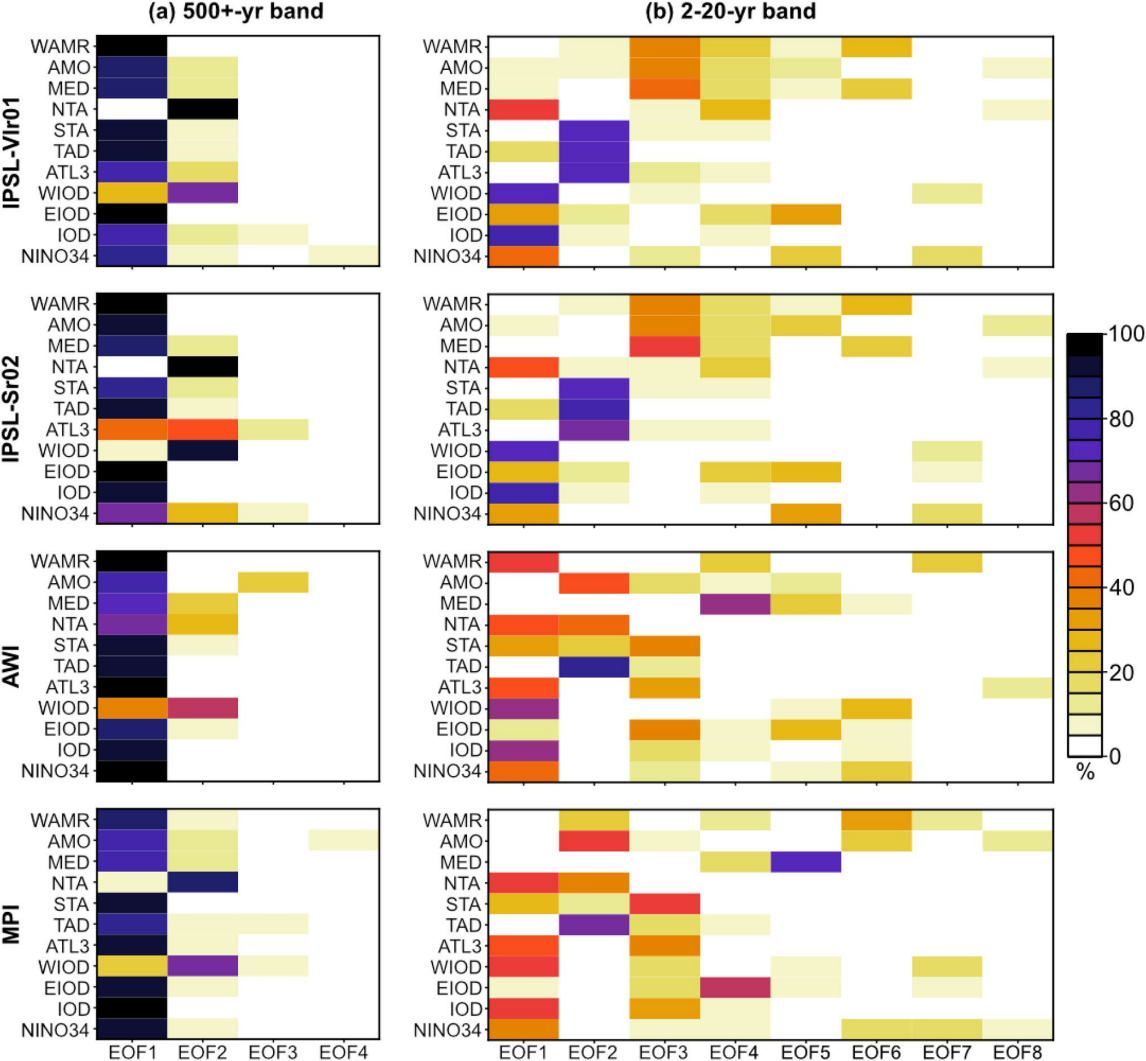
Variance of the WAMR and SST indices explained by the first (**a**) 4 EOF modes filtered in the 500‐year low‐pass band and (**b**) 8 EOF modes filtered in the 2-20-year band for each transient simulation. The abbreviations in the x-axis are detailed in Table 2

EOF1 explains ca 70% of the variability and EOF2 ca 20% of the variability of the WAMR–SST correlation matrix in all of the models (Fig. 3a). However, the first EOF explains ~ 90% of the overall WAMR variance, while the EOF2 contributes only 5% at most to this variability (Fig. 4a).

**Fig. 5:**
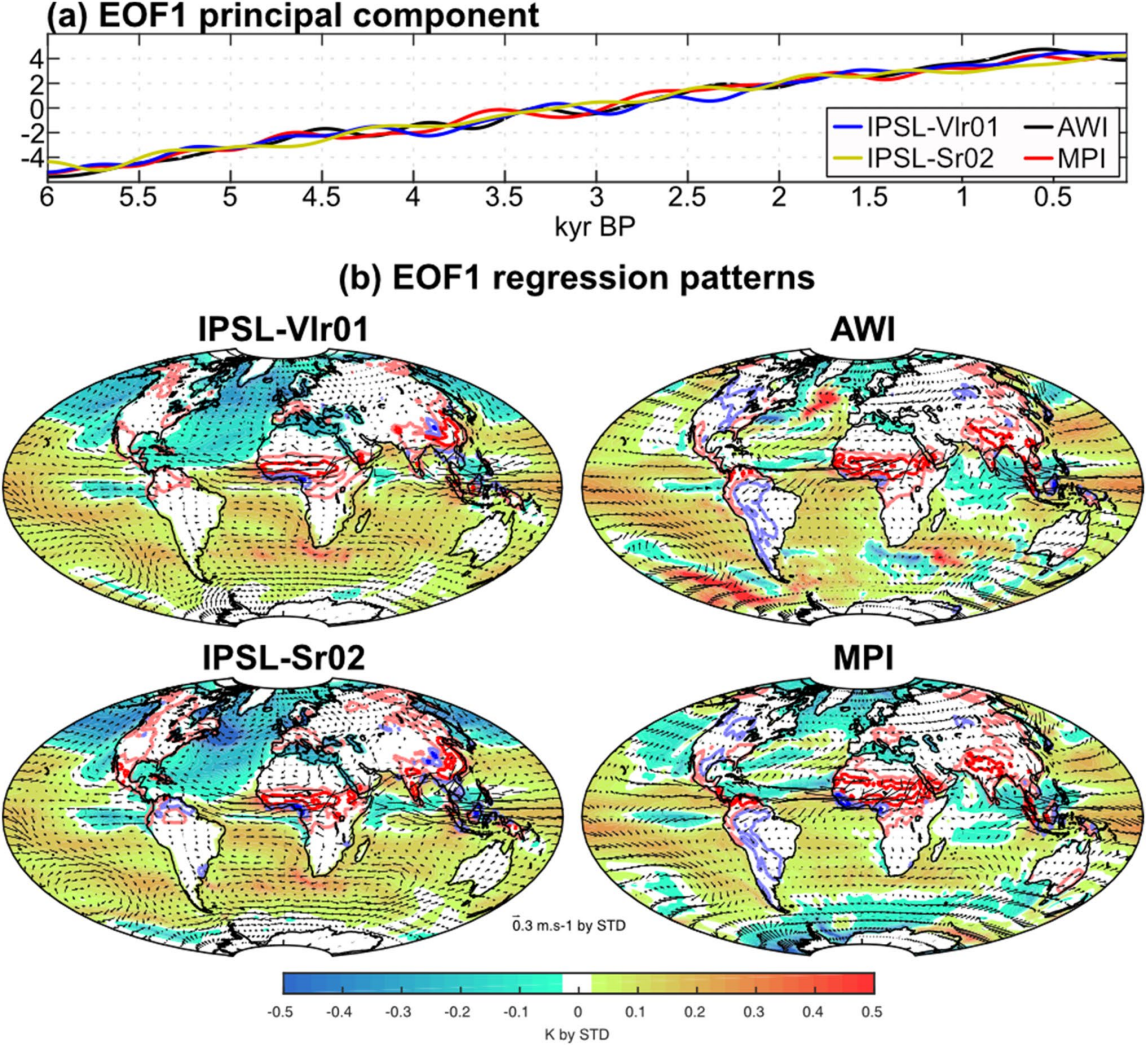
**a** Gradual orbitally-driven changes in WAMR, SST and 850 hPa atmospheric circulation from mid- to late Holocene. The timeseries correspond to the EOF1’s principal component of the 500-year low-pass filtered WAMR – SST correlation matrix for the four transient simulations. **b** Associated anomalous patterns in SST (K, shadings), 850‐hPa wind (m/s, arrows) and rainfall over land (red/blue contours for dry/wet anomalies, with light and dark colors for |0.1| and |0.5| mm/d anomalies, respectively). The patterns are obtained by regressing the 500-year low-pass filtered anomalies onto the EOF1 principal component

EOF1 shows a gradual and near linear trend of SST and WAMR over the past 6,000 years that is consistent between the models (Fig. 5a) and consistent with the long-term precession-induced changes (Fig. 1a). The regression patterns of rainfall, 850-hPa wind and SST onto the EOF1 principal component (Fig. 4b) reflect the gradual weakening of the interhemispheric SST gradient and land-sea contrast during boreal summer resulting in a southward shift of the ITCZ, a weakening of the monsoon circulation and a strong decrease of the global monsoon, especially in Asia and West Africa. The four transient simulations provide a similar picture of these changes (Fig. 5b). They also show consistency in the contribution of different SST indices with AMO, MED, STA, TAD, EIOD, IOD and NINO3.4. These SST regions have strong contributions to EOF1 in all models (Fig. 4a). The NTA region does not contribute significantly to EOF1 (except for the AWI model; Fig. 4a), suggesting this region may not be critical to explain the long-term trend in WAMR.

However, there are some differences between the models, especially in the North Atlantic and West Africa. The two IPSL simulations show a widespread SST cooling and a weak reduction of the 850-hPa anticyclonic circulation in the North Atlantic, while the AWI and MPI models have a dipole-like SST pattern associated with marked weakening of the 850-hPa anticyclonic circulation (Fig. 5b). The drying trend in West Africa is more extensive in the AWI and MPI than in the two IPSL simulations, a difference which reflects the fact that the mid-Holocene and present-day West African rain band is too far south in these IPSL models (Hourdin et al. 2013; Braconnot et al. 2019a). The similarities between the AWI and MPI models likely reflect the fact that they have the same atmospheric component. The evolution of the equatorial Atlantic cold tongue also differs between the models, becoming stronger from the mid-Holocene onwards in the IPSL simulations and weaker in the AWI and MPI simulations. The evolution of ATL3 SST is slightly more complex in the IPSL-Vlr01 simulation and particularly in the IPSL-Sr02 simulation than in the AWI and MPI simulations, being distributed across several EOFs in the IPSL simulations. Such differences in model behaviour probably reflect difficulties in simulating Bjerknes feedback associated with biases in atmospheric circulation over the equatorial Atlantic (Voldoire et al. 2019).

The regression patterns of rainfall, 850-hPa wind and SST onto the principal component of EOF2 show that changes are largely confined to the tropical belt (Fig. S4). The NTA and WIOD have strong contributions to EOF2 across all models (Fig. 4a). In the IPSL simulations, the pattern tends to amplify the warming in the northern hemisphere from 6,000 to 4,500 BP and to dampen the cooling during the last 1,500 years, a temporal evolution that has been attributed to the response to greenhouse gas forcing (Braconnot et al. 2019b). The pattern is noisier in the AWI and MPI simulations but the temporal evolution is similar to that in the IPSL simulations (Fig. S4). This indicates that the atmospheric pattern of the response to greenhouse gases projects better onto the pattern of the response to the long-term insolation in the MPI and AWI models (which share the same atmospheric component), so that the second EOF modulates the pattern in different regions from those affected in the IPSL simulations.

#### Interannual-to-decadal variability

The WAMR–SST relationship is more complex on the interannual-to-decadal timescale, with EOF1 explaining only 25–30% of the variance of the WAMR–SST correlation matrix (Fig. 3b) and the first 7 EOFs explaining each more than 10% of the WAMR variance (Fig. 4b). EOF modes are different from one model to another by construction due to the orthogonal constraints. The main consequence is that similar patterns among the models would not necessarily show up on the same EOF. This explains why there is no consistency about which EOFs explain a significant fraction of the WAMR variance across the four models (Fig. 4b). In addition, differences also arise due to model dependency in simulating the pattern and magnitude of modes of variability. The AWI and MPI models behave differently despite using the same atmospheric component, while the two IPSL simulations give broadly similar results despite the different representation of land hydrology, snow and vegetation (Fig. 4b).

These differences are further illustrated by comparing the EOF mode associated with the largest WAMR variance. In the two IPSL models, EOF3 explains 40% of the WAMR variance and is strongly connected to the AMO and Mediterranean Sea SSTs (Fig. 4b). In the AWI simulation, EOF1 explains ~ 50% of the WAMR variance and 30 to 50% of the variance of tropical SSTs (Fig. 4b). This EOF mode, which represents ENSO – Atlantic teleconnections and their potential impact on WAMR variability, only contributes significantly to WAMR variability in the AWI simulation (Fig. 4b). This probably reflects the fact that ENSO – Atlantic teleconnections are poorly captured by most current climate models (Zhao et al. 2007; Joly et al. 2007; Wang et al. 2014). In the MPI simulation, EOF6 explains ~ 30% of the WAMR variance and 20% of the AMO and NINO3.4 SST variance (Fig. 2b).

**Fig. 6:**
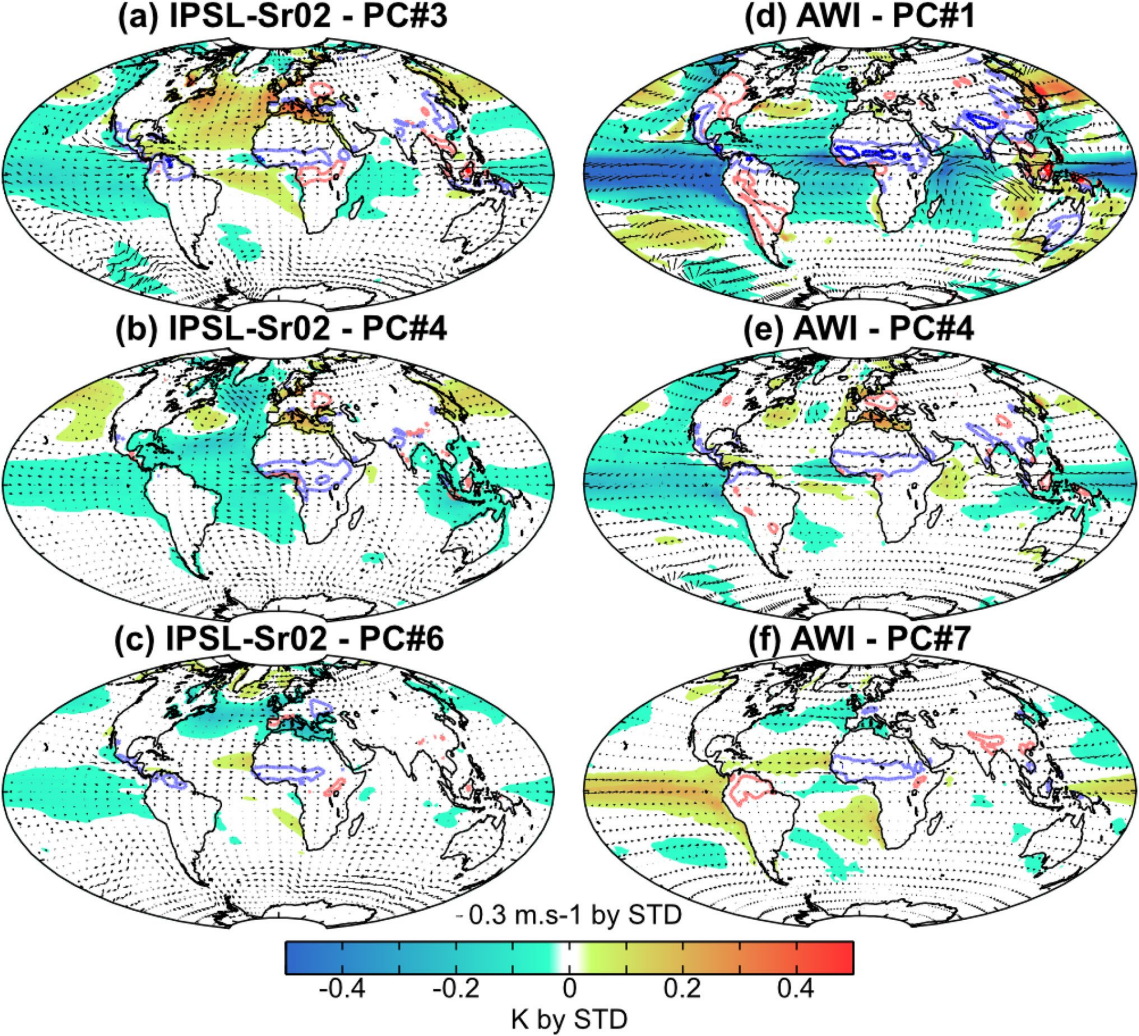
Modes of interannual‐to‐decadal variability accounting for WAMR for the last 6000 years. The patterns are obtained by regressing 2-20-year SST (K, shadings), 850‐hPa wind (m/s, arrows) and rainfall over land (red/blue contours for dry/wet anomalies, with light and dark colors for |0.1| and |0.5| mm/d anomalies, respectively) onto EOF modes explaining at least 10% of the WAMR interannual-to-decadal variability. **a**–**c** Patterns associated with EOFs 3, 4 and 6 for the IPSL-Sr02 simulation, respectively. Note that patterns in (**a**–**c**) are similar for the IPSL-Vlr01 simulation, hence not shown. **d**–**f** Patterns associated with EOFs 1, 4 and 7 for the AWI simulation, respectively

**Fig. 7:**
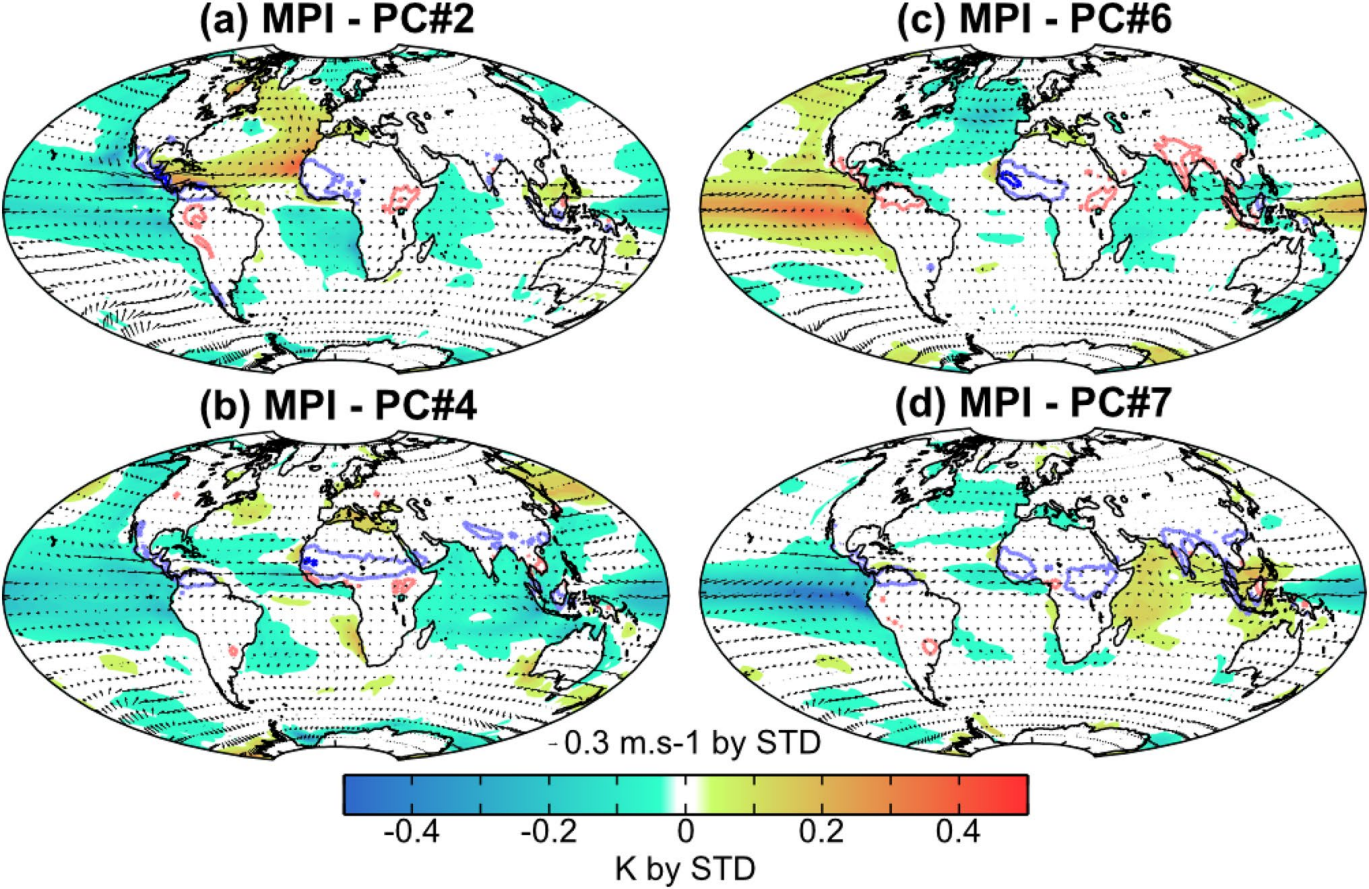
Same as Fig. 5 but for (**a**–**d**) EOFs 2, 4, 6 and 7 of the MPI simulation, respectively

Differences between models can also be seen in the regression patterns associated with the EOF modes accounting for a significant fraction of the WAMR variance (Figs. 6, 7). Wet WAMR anomalies are associated with wetter conditions across the whole of the Sahel in the IPSL and AWI simulations (Fig. 6), but wet WAMR anomalies occur with dry anomalies in East Africa in the MPI simulation (Fig. 7). Wet WAMR anomalies systematically occur under La Niña-like conditions in the IPSL simulations (Fig. 6a–c) but can occur under El Niño-like conditions in the AWI (Fig. 6f) and MPI (Fig. 7c) simulations.

Although wet WAMR anomalies can be produced in multiple ways in each of the models (Figs. 6–7), there are similarities between the models in many of the associated changes in ocean modes. Three main inter-basin configurations are associated with wet WAMR anomalies among the models:

1. Strong La Niña co-occurring with IOD-, AMO + and warm SST anomalies in the Mediterranean Sea (Fig. 6a). This configuration is similar to the pattern shown by AWI-EOF4 in the Pacific and Mediterranean Sea (Fig. 6e), and closely resembles the pattern in MPI-EOF2 (Fig. 7a).
2. Strong La Niña co-occurring with IOD + , AMO- and warm SST anomalies in the Mediterranean Sea (Fig. 6b). This configuration is similar to the patterns in the North Atlantic and the Mediterranean Sea shown in AWI-EOF1 (Fig. 6d), although the SST anomalies in the Mediterranean Sea are weaker in AWI. This configuration also appears in MPI-EOF4 (Fig. 7b), although the AMO- pattern is weaker than in the IPSL models, and in MPI-EOF6 (Fig. 7c) although the SST anomalies in the Pacific are of opposite sign to those in IPSL.
3. Weak La Niña co-occurring with cold SST anomalies in the AMO region and the Mediterranean Sea and near normal Indian Ocean SSTs (Fig. 6c). This configuration is similar to the AWI-EOF7 in the Atlantic (Fig. 6f), although again SST anomalies in the Pacific are of opposite sign from those in IPSL This configuration is also similar to MPI-EOF7 (Fig. 7d), although the MPI model has warm SST anomalies in the IOD whereas the IPSL simulations show little change in SSTs.

Overall, the differences between the models in the relative importance of different EOF modes for monsoon variability, the strength of the contribution of different ocean modes to wet WAMR anomalies, and the broader spatial patterns of rainfall change associated with wet WAMR anomalies, all highlight the fact that the causes of monsoon variability on interannual-to-decadal timescales are more complex than those that drive monsoon changes on orbital timescales. Nevertheless, certain common features emerge. In particular, it is clear that on this timescale several different configurations of changes in regional SSTs can have a similar impact on changes in monsoon rainfall. Furthermore, in all of the models, the impact of SST changes in one region, for example in the Mediterranean Sea or in the tropical Atlantic, is strongly modulated by more far-field changes. This complexity reflects the fact that variability on these timescales is highly stochastic in nature and there are no straight forward explanations for the short-term variability in the WAMR, even in the context of long-term, externally forced, climate changes. Thus, there are limitations to the predictability of WAMR rainfall on timescales that might be relevant for management and adaptation.

### Tracking multi-centennial oscillation and trend of WAMR interannual-to-decadal variability

**Fig. 8:**
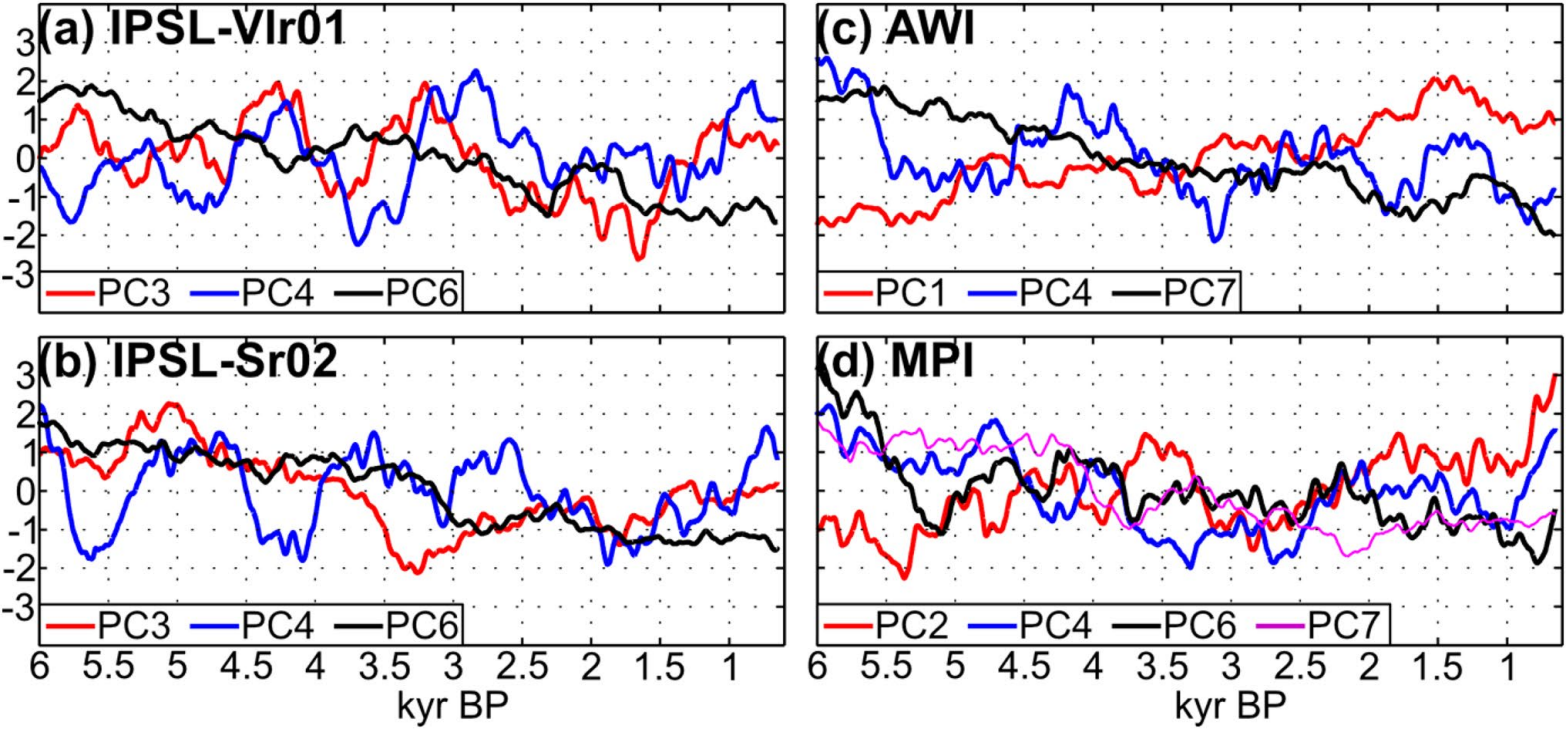
Multi-centennial modulation of WAMR interannual-to-decadal variability for the (**a**) IPSL-Vlr01, (**b**) IPSL-Sr02, (**c**) AWI and (**d**) MPI simulations. Each curve shows the 500-yr running standard deviation of the standardized Principal Component (PC) associated with the EOF modes explaining at least 10% of the WAMR inter-annual-to-decadal variability (see Figs. 6, 7 for the regression pattern associated with these EOF modes). A 50-yr moving average is applied to smooth out high frequency variability

The EOF framework provides a simple way to identify modes of variability contributing to multi-centennial oscillations and long-term trend of the WAMR interannual-to-decadal variability. Analysis of the PC timeseries of the EOF modes explaining more than 10% of the WAMR interannual-to-decadal variability (Fig. 8) provides insight into the modes contributing to the multi-centennial oscillations in WAMR variability and its overall decreasing trend.

The multi-centennial modulations of the WAMR variability are reflected in EOF3 for the IPSL simulations, EOF4 for the AWI simulation and EOF2 and EOF4 for the MPI simulation (Fig. 8 and Table 3). These EOF modes are characterised by La Niña / PDO- conditions in the Pacific and a weakening and/or poleward shift of subtropical high-pressure systems over the Atlantic during wet WAMR seasons (Figs. 6, 7), which acts to reduce trade winds along the equatorial Atlantic and favor moisture advection into West Africa.

**Table 3:** Bravais Pearson correlation between WAMR interannual-to-decadal variability smoothed along 500-yr moving windows and that of the principal component timeseries of the EOF modes contributing to at least 10% of the WAMR variance for the last 6,000 years

	IPSL-Vlr01	IPSL-Sr02	AWI	MPI
EOF1	/	/	− 0.44	/
EOF2	/	/	/	− 0.39
EOF3	0.67	0.77	/	/
EOF4	− 0.03	0.32	0.36	0.61
EOF6	0.80	0.83	/	0.70
EOF7	/	/	0.73	0.82

The decreasing trend in WAMR variability is mainly associated with EOF6 in the IPSL simulations (Fig. 8a, b), EOF1 and EOF7 in the AWI simulation (Fig. 8c) and EOF6 and EOF7 in the MPI simulation (Fig. 8d). There is a strong positive relationship between the variability of these EOF modes and that of WAMR, with correlation values between 0.7 and 0.8 (Table 3), except for EOF1 in the AWI simulation which is negatively correlated with the WAMR. The variability of the AWI-EOF1 mode, which reflects increased ENSO variability with time, is negatively correlated with WAMR variability (r = − 0.44; Table 3) indicating that the influence of ENSO on WAMR variability weakened from mid- to late Holocene in this simulation even though the strength of ENSO increased. The EOF modes showing a positive correlation with WAMR in the different simulations reflect different SST and circulation patterns in the Pacific and Indian Oceans, but they all have anomalously cold SSTs in the North Atlantic and the Gulf of Guinea and anomalously warm SSTs west of West Africa during wet WAMR seasons (Figs. 6 and 7). We can thus deduce that the Atlantic part of the global pattern is the one that is key for the WAMR variability trend, despite the fact that it is associated with different patterns in the other basins. These anomalously cold or warm conditions weaken or become less frequent with time as the boreal summer interhemispheric energy gradient is reduced in response to orbital forcing. Despite different model biases, all the simulations show similar linkages between the trend in the climate mean state and the trend in interannual-to-decadal variability in West Africa.

## Discussion and conclusions

This study has examined the consistency of mid- to late Holocene changes in West African monsoon rainfall (WAMR) and its driving modes in four 6,000-yr long transient simulations. At the millennial timescale, simulated changes are very consistent among the models. WAMR shows a strong and steady reduction from mid- to late Holocene forced by a decrease in the interhemispheric thermal gradient caused by orbital changes. This is a robust result across these simulations, and is consistent with the influence of the interhemispheric thermal gradient on present-day variations in monsoon intensity (Biasutti et al. 2008; Xue et al. 2022). However, this gradual reduction in the WAMR would appear to be inconsistent with evidence of an abrupt termination of humid conditions at ca 5 ka adduced from abrupt increases in dust in marine cores from around northern Africa (DeMenocal et al. 2000; McGee et al. 2013; Tierney and DeMenocal 2013). Records from the continental interior are often of low temporal resolution or discontinuous. Nevertheless, these records are consistent with a regionally time-transgressive but progressive aridification of northern Africa through the late Holocene (Gasse 2000; Hoelzmann et al. 2004; Cremaschi et al. 2006; Kröpelin et al. 2008; Francus et al. 2013; Shanahan et al. 2015), as shown by the simulations. Indications of abrupt drying at individual sites, such as the drying of Lake Mega-Chad at ca 5 ka (Armitage et al. 2015), probably reflect the fact that multi-decadal to multi-centennial droughts are superimposed on this long term aridification trend and the progressive southward positioning of the rain belt means there is no subsequent recovery. Certainly, lake and pollen records from further south show a progressive aridification through the late Holocene (Shanahan et al. 2015; Lemmonier and Lézine 2022).

The strong and steady WAMR reduction simulated from mid- to late Holocene by the four transient simulations would also appear to be inconsistent with recent modelling studies suggesting abrupt rainfall changes in West Africa through changes in vegetation, and associated albedo feedbacks, or in the Atlantic Meridional Overturning Circulation (AMOC; Menviel et al. 2021; Hopcroft and Valdes 2022; Li et al. 2023). There are several possible reasons for this apparent inconsistency. Firstly, we are examining trends averaged over a large area (7–18°N by 15°W–20°E); it is possible that some parts of this domain exhibited more rapid shifts. In the HADCM3 transient simulations (Hopcroft and Valdes 2022), for example, abrupt changes in rainfall occur between 20°N and 30°N. Small but persistent changes in rainfall in this region can lead to significant changes in vegetation, which feedback on the water cycle. Our simulations also show more abrupt shifts in rainfall in this more northern region (Fig. S3). Secondly, our focus is on the trend in monsoon rainfall during the last 6 kyr. The abrupt shifts documented by Hopcroft and Valdes (2022) mostly occur between 8 and 6 kyr BP. Finally, the multivariate EOF analysis approach is designed to assess multiscale changes in WAMR variability rather than abrupt shifts, which are better identified using other methods such as the KDJ index (Li et al. 2023) or information entropy (Falasca et al. 2020).

We cannot rule out that the apparent lack of abrupt changes in the WAMR reflects model deficiencies in capturing the AMOC (e.g. Heuzé, 2017), as suggested by Menviel et al. (2021). The AMO shows a persistent cooling trend driven by orbital changes in all our simulations (Fig. 1b). The AMO cooling trend is in line with a gradual weakening of the AMOC during the Holocene suggested by some studies (Thornalley et al. 2018; Caesar et al. 2021; Jomelli et al. 2022), but at odds with Jiang et al. (2023) who found no consistent trend in overall AMOC strength during the mid-to-late Holocene in an ensemble of nine transient simulations. Furthermore, none of the four simulations have abrupt changes in the AMO at the interannual-to-decadal timescale, although there are multi-centennial modulations in the interannual-to-decadal variability of the AMO that clearly influence WAMR variability (Fig. 2a, b).

The models all simulate decreased variability of the WAMR on the interannual-to-decadal timescale during the Holocene. This decrease in interannual-to-decadal variability contrasts with the increased variability of the Indian summer monsoon superimposed on a similar long-term drying trend (Crétat et al. 2020). The ocean–atmosphere modes involved, as well as the timing and amplitude of changes, vary between the models. WAMR interannual-to-decadal variability strongly relates to ENSO in the AWI simulation, to the Mediterranean Sea in the two IPSL simulations and to the AMO and tropical Atlantic dipole in the MPI simulation. These modes are consistent with those influencing WAMR variability in the recent period. The inter-model differences reflect the fact that these are complex inter-basin teleconnections that are sensitive to differences in the physical parametrizations between models and may also relate to the mixed influence of orbital forcing and internal variability and to intrinsic model biases in simulating WAMR and its teleconnection patterns (e.g. Joly et al. 2007). They may also result from our area-averaged index approach (same indices among the models and throughout the Holocene), which is much stricter compared to spatial pattern approaches (e.g., Maximum Covariance Analysis in Joly et al. 2007) and time-varying indices, but much easier to handle and interpret.

Our results point also out multi-centennial modulations of the WAMR which are consistent with several records both from the central Sahara (e.g. Cremaschi et al. 2006; Van der Meeren et al. 2022) and from locations outside but adjacent to the Sahara (e.g. Street-Perrott et al. 2000; Nguetsop et al. 2013; Zielhofer et al. 2019). The comparative lack of tree ring or speleothem records from northern Africa (see e.g. Braun et al. 2019; Gebrekirstos et al. 2014), which could provide records of annual to decadal variability, currently precludes an analysis of the realism of the simulated decrease in short-term variability over the Holocene. Furthermore, such a comparison might not be warranted because the simulations examined here do not include all natural forcings, such as solar forcing, volcanic activity and aerosols, that might influence the short-term variability.

Finally, this study reveals a complex relationship between mean state changes and interannual-to-decadal variability changes from mid- to late Holocene. The twenty-first century climate simulations project an increase in both the mean state and the interannual variability of monsoon rainfall over West Africa (Akinsanola et al. 2020), India (Katzenberger et al. 2021) and, more generally, increased rainfall interannual variability in the tropics (He and Li 2019). The future projections are predominantly driven by thermodynamical changes, while dynamical changes dominate in the Holocene (D’Agostino et al. 2020). Although Holocene and 21^st^ climate are not analogues, our work suggests that multi-centennial oscillations in interannual-to-decadal variability are independent of orbital changes but can lead to strong compound events. How such low frequency modulations of interannual variability could change under twenty-first century global warming is an important but open question in West Africa to foster water resource management and planning. As a result, analyzing the extent to which rainfall variability may change under global warming should not be neglected.

### Supplementary Information

Below is the link to the electronic supplementary material.

Supplementary file1 (DOCX 740 KB)

## Data Availability

The transient simulations were run as part of the JPI-Belmont project PACMEDY. The IPSL, AWI and MPI simulations are available upon reasonable request to *pascale.braconnot@lsce.ipsl.fr*, *gerrit.Lohmann@awi.de* and *johann.jungclaus@mpimet.mpg.de*, respectively.
