# Supplementary material for: Orbitally forced and internal changes in West African rainfall interannual-to-decadal variability for the last 6000 years
Source: Clim Dyn. 2023 Nov 30;62(3):2301–16. doi: 10.1007/s00382-023-07023-y (PMC10899366; doi:10.1007/s00382-023-07023-y)
Supplement: Supplementary file 1 — Supplementary file1 (DOCX 740 KB) [file 382_2023_7023_MOESM1_ESM.docx]

**Supplementary material: Orbitally forced and internal changes in West African rainfall interannual-to-decadal variability for the last 6000 years**

This supplementary contains descriptions of the models used for the transient simulations and two additional figures.

**Description of the IPSL-CM-TR5AS model**

The IPSL-Vlr01 simulation was run with the IPSL-CM-TR5AS model, an updated version of the IPSLCM5A-LR model used for PMIP3-CMIP5 (Dufresne et al., 2013; Kageyama et al., 2013) with improved climatology and computing performance (Sepulchre et al., 2020). Sea ice is run at the ocean horizontal resolution and land surface at the atmospheric horizontal resolution. The carbon cycle over land and ocean is fully interactive. However, atmospheric CO2 concentration is prescribed, as is vegetation type. The transient simulation was begun from last year of a mid-Holocene simulation that was run following the PMIP4 mid-Holocene protocol for orbital and greenhouse gas forcing (Otto-Bliesner et al., 2017). Results from the IPSL-Vlr01 simulation have been discussed in Braconnot et al. (2019a).

**Description of the IPSL-CM-TR6AV**

The IPSL-Sr02 simulation was run with the IPSL-CM-TR6AV model, an updated version of the IPSLCM5A-MR model (Dufresne et al., 2013) in which the land surface component has been substantially modified to include a 11 layer hydrology, a new snow model and dynamical vegetation (Braconnot et al., 2019b). This model version couples the LMDZ atmospheric model with 144 × 142 grid points in latitude and longitude (2.5° × 1.27°) and 39 vertical levels (Hourdin et al., 2013) to the ORCA2 ocean model at 2° resolution (Madec, 2008). The ocean biogeochemical model PISCES is also coupled to the ocean physics and dynamics to represent marine biochemistry and the carbon cycle (Aumont and Bopp, 2006). The land surface scheme is based on the ORCHIDEE model (Krinner et al., 2005), which includes a mosaic vegetation representation in each grid box, based on 13 plant functional types (PFTs), and has an interactive carbon cycle (Krinner et al., 2005). The land surface model has been updated by inclusion of a 11- layer physically based hydrological scheme (de Rosnay et al., 2002) to replace the original 2-layer bucket-type hydrology (Ducoudré et al., 1993). It also includes a new prognostic 3-layer snow model (Wang et al., 2013). ORCHIDEE includes a river runoff scheme to route runoff to the river mouths or to coastal areas (d’Orgeval et al., 2008) and a scheme for routing ice melt to the ocean (Marti et al., 2010).

**Description of the AWI-ESM2 model**

AWI-ESM2 is an extension of the AWI climate model version 2 (AWI-CM2) (Sidorenko et al., 2019; https://fesom.de/models/awi-esm/). The atmospheric module is represented by the general circulation model ECHAM6 (Stevens et al., 2013), here run at T63 horizontal resolution (~180 km). The ocean and sea-ice model is based on FESOM2 (Danilov et al., 2017) and is based on the finite volume discretization formulated on unstructured meshes. The spatial resolution for the ocean is up to 15 km over polar and coastal regions, and 135 km for far-field oceans, with 46 uneven vertical depths. AWI-ESM2 includes the land surface model JSBACH, which is based on a tiling of the land surface and includes dynamic vegetation with 12 plant functional types and two types of bare surface (Reick et al., 2013). This model has been evaluated under modern climate conditions (Sidorenko et al., 2019) and has been used in a number of applications (e.g., Lohmann et al., 2020; Vorrath et al., 2020; Kageyama et al., 2021; Otto-Bleisner et al., 2021; Lamping et al., 2021).

**Description of the MPI-ESM model**

The MPI-ESM includes the atmospheric model ECHAM6 (Stevens et al., 2013). The ocean module is MPI-OM (Jungclaus et al., 2013), which includes a sea-ice model and the biogeochemical chemistry model HAMOCC (Ilyina et al., 2013). The land-surface module, JSBACH (Reick et al., 2013) includes dynamic vegetation. The model version applied here includes several updates and corrections compared to the CMIP5 version (Giorgetta et al., 2013) and is identical to the model used in the MPI “Grand Ensemble” project (Maher et al., 2019). The Holocene transient simulation corresponds to the simulation with dynamical vegetation presented in Dallmeyer et al. (2021) and was begun after a 3000 year spin-up, with constant boundary conditions fixed at 7950 BP (Dallmeyer et al., 2021).

**Model References**

Aumont O, Bopp L (2006) Globalizing results from ocean in situ iron fertilization studies: GLOBALIZING IRON FERTILIZATION. Global Biogeochem Cycles 20:n/a-n/a. <https://doi.org/10.1029/2005GB002591>

Braconnot P, Crétat J, Marti O, et al (2019a) Impact of Multiscale Variability on Last 6,000 Years Indian and West African Monsoon Rain. Geophys Res Lett 46:14021–14029. <https://doi.org/10.1029/2019GL084797>

Braconnot P, Zhu D, Marti O, Servonnat J (2019b) Strengths and challenges for transient Mid- to Late Holocene simulations with dynamical vegetation. Clim Past 15:997–1024. <https://doi.org/10.5194/cp-15-997-2019>

d’Orgeval T, Polcher J, de Rosnay P (2008) Sensitivity of the West African hydrological cycle in ORCHIDEE to infiltration processes. Hydrol Earth Syst Sci 12:1387–1401. <https://doi.org/10.5194/hess-12-1387-2008>

Dallmeyer A, Claussen M, Lorenz SJ, et al (2021) Holocene vegetation transitions and their climatic drivers in MPI-ESM1.2. Clim Past 17:2481–2513. <https://doi.org/10.5194/cp-17-2481-2021>

Danilov S, Sidorenko D, Wang Q, Jung T (2017) The Finite-volumE Sea ice–Ocean Model (FESOM2). Geosci Model Dev 10:765–789. <https://doi.org/10.5194/gmd-10-765-2017>

de Rosnay P, Polcher J, Bruen M, Laval K (2002) Impact of a physically based soil water flow and soil-plant interaction representation for modeling large-scale land surface processes: PHYSICALLY BASED SOIL HYDROLOGY IN GCM. J Geophys Res 107:ACL 3-1-ACL 3-19. <https://doi.org/10.1029/2001JD000634>

Ducoudré NI, Laval K, Perrier A (1993) SECHIBA, a New Set of Parameterizations of the Hydrologic Exchanges at the Land-Atmosphere Interface within the LMD Atmospheric General Circulation Model. J Climate 6:248–273. [https://doi.org/10.1175/1520-0442(1993)006<0248:SANSOP>2.0.CO;2](https://doi.org/10.1175/1520-0442(1993)006%3c0248:SANSOP%3e2.0.CO;2)

Dufresne J-L, Foujols M-A, Denvil S, et al (2013) Climate change projections using the IPSL-CM5 Earth System Model: from CMIP3 to CMIP5. Clim Dyn 40:2123–2165. <https://doi.org/10.1007/s00382-012-1636-1>

Giorgetta MA, Jungclaus J, Reick CH, et al (2013) Climate and carbon cycle changes from 1850 to 2100 in MPI-ESM simulations for the Coupled Model Intercomparison Project phase 5: Climate Changes in MPI-ESM. J Adv Model Earth Syst 5:572–597. <https://doi.org/10.1002/jame.20038>

Hourdin F, Grandpeix J-Y, Rio C, et al (2013) LMDZ5B: the atmospheric component of the IPSL climate model with revisited parameterizations for clouds and convection. Clim Dyn 40:2193–2222. <https://doi.org/10.1007/s00382-012-1343-y>

Ilyina T, Six KD, Segschneider J, et al (2013) Global ocean biogeochemistry model HAMOCC: Model architecture and performance as component of the MPI‐Earth system model in different CMIP5 experimental realizations. J Adv Model Earth Syst 5:287–315. <https://doi.org/10.1029/2012MS000178>

Jungclaus JH, Fischer N, Haak H, et al (2013) Characteristics of the ocean simulations in the Max Planck Institute Ocean Model (MPIOM) the ocean component of the MPI‐Earth system model. J Adv Model Earth Syst 5:422–446. <https://doi.org/10.1002/jame.20023>

Kageyama M, Braconnot P, Bopp L, et al (2013) Mid-Holocene and Last Glacial Maximum climate simulations with the IPSL model—part I: comparing IPSL_CM5A to IPSL_CM4. Clim Dyn 40:2447–2468. <https://doi.org/10.1007/s00382-012-1488-8>

Kageyama M, Harrison SP, Kapsch M-L, et al (2021) The PMIP4 Last Glacial Maximum experiments: preliminary results and comparison with the PMIP3 simulations. Clim Past 17:1065–1089. <https://doi.org/10.5194/cp-17-1065-2021>

Krinner G, Viovy N, de Noblet-Ducoudré N, et al (2005) A dynamic global vegetation model for studies of the coupled atmosphere-biosphere system: DVGM FOR COUPLED CLIMATE STUDIES. Global Biogeochem Cycles 19:. <https://doi.org/10.1029/2003GB002199>

Lamping N, Müller J, Hefter J, et al (2021) Evaluation of lipid biomarkers as proxies for sea ice and ocean temperatures along the Antarctic continental margin. Clim Past 17:2305–2326. <https://doi.org/10.5194/cp-17-2305-2021>

Lohmann G, Butzin M, Eissner N, et al (2020) Abrupt Climate and Weather Changes Across Time Scales. Paleoceanography and Paleoclimatology 35:. <https://doi.org/10.1029/2019PA003782>

Madec G (2008) NEMO ocean engine, available at: https://www. nemo-ocean.eu/doc/ (last access: 4 March 2023).

Maher N, Milinski S, Suarez‐Gutierrez L, et al (2019) The Max Planck Institute Grand Ensemble: Enabling the Exploration of Climate System Variability. J Adv Model Earth Syst 11:2050–2069. <https://doi.org/10.1029/2019MS001639>

Marti O, Braconnot P, Dufresne J-L, et al (2010) Key features of the IPSL ocean atmosphere model and its sensitivity to atmospheric resolution. Clim Dyn 34:1–26. <https://doi.org/10.1007/s00382-009-0640-6>

Otto-Bliesner BL, Braconnot P, Harrison SP, et al (2017) The PMIP4 contribution to CMIP6 – Part 2: Two interglacials, scientific objective and experimental design for Holocene and Last Interglacial simulations. Geosci Model Dev 10:3979–4003. <https://doi.org/10.5194/gmd-10-3979-2017>

Otto-Bliesner BL, Brady EC, Zhao A, et al (2021) Large-scale features of Last Interglacial climate: results from evaluating the &lt;i&gt;lig127k&lt;/i&gt; simulations for the Coupled Model Intercomparison Project (CMIP6)–Paleoclimate Modeling Intercomparison Project (PMIP4). Clim Past 17:63–94. <https://doi.org/10.5194/cp-17-63-2021>

Reick CH, Raddatz T, Brovkin V, Gayler V (2013) Representation of natural and anthropogenic land cover change in MPI-ESM: Land Cover in MPI-ESM. J Adv Model Earth Syst 5:459–482. <https://doi.org/10.1002/jame.20022>

Sepulchre P, Caubel A, Ladant J-B, et al (2020) IPSL-CM5A2 – an Earth system model designed for multi-millennial climate simulations. Geosci Model Dev 13:3011–3053. <https://doi.org/10.5194/gmd-13-3011-2020>

Shi X, Werner M, Krug C, et al (2022) Calendar effects on surface air temperature and precipitation based on model-ensemble equilibrium and transient simulations from PMIP4 and PACMEDY. Clim Past 18:1047–1070. <https://doi.org/10.5194/cp-18-1047-2022>

Sidorenko D, Goessling HF, Koldunov NV, et al (2019) Evaluation of FESOM2.0 Coupled to ECHAM6.3: Preindustrial and HighResMIP Simulations. J Adv Model Earth Syst 11:3794–3815. <https://doi.org/10.1029/2019MS001696>

Stevens B, Giorgetta M, Esch M, et al (2013) Atmospheric component of the MPI‐M Earth System Model: ECHAM6. J Adv Model Earth Syst 5:146–172. <https://doi.org/10.1002/jame.20015>

Vorrath M-E, Müller J, Rebolledo L, et al (2020) Sea ice dynamics in the Bransfield Strait, Antarctic Peninsula, during the past 240 years: a multi-proxy intercomparison study. Clim Past 16:2459–2483. <https://doi.org/10.5194/cp-16-2459-2020>

Wang T, Ottlé C, Boone A, et al (2013) Evaluation of an improved intermediate complexity snow scheme in the ORCHIDEE land surface model: ORCHIDEE SNOW MODEL EVALUATION. J Geophys Res Atmos 118:6064–6079. <https://doi.org/10.1002/jgrd.50395>

**
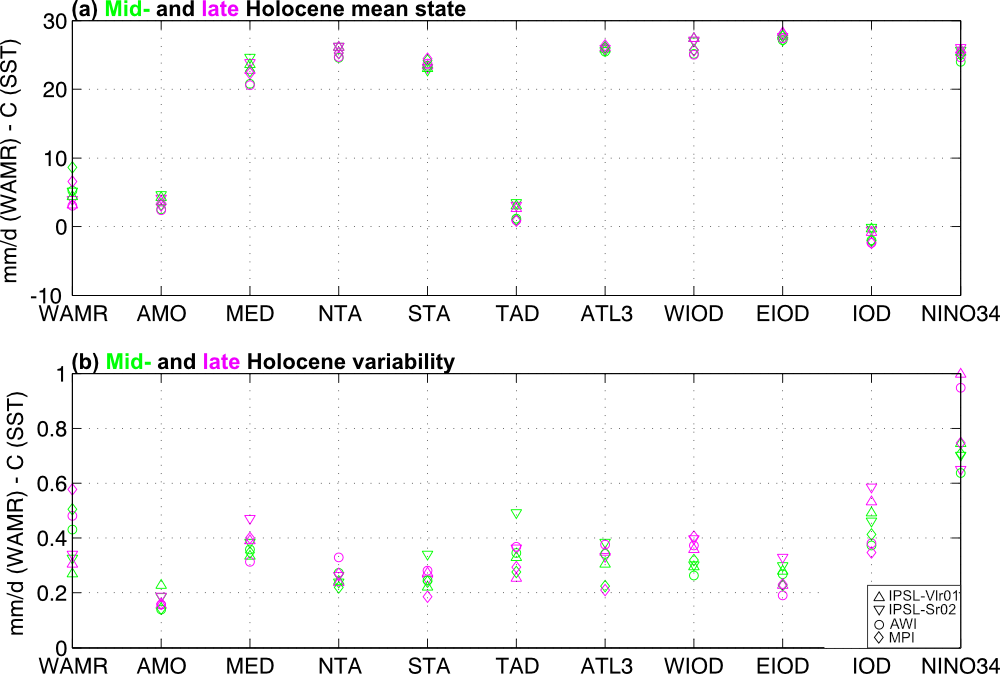
**

**Figure S1** Mid- and late Holocene (a) mean WAMR and SST conditions and (b) associated variability. The mean conditions are computed along the first and last 500 years of the 6000-yr long simulations. The variability is defined as the mean standard deviation computed on 20-yr chunks for each of the two 500 year periods (25 chunks).


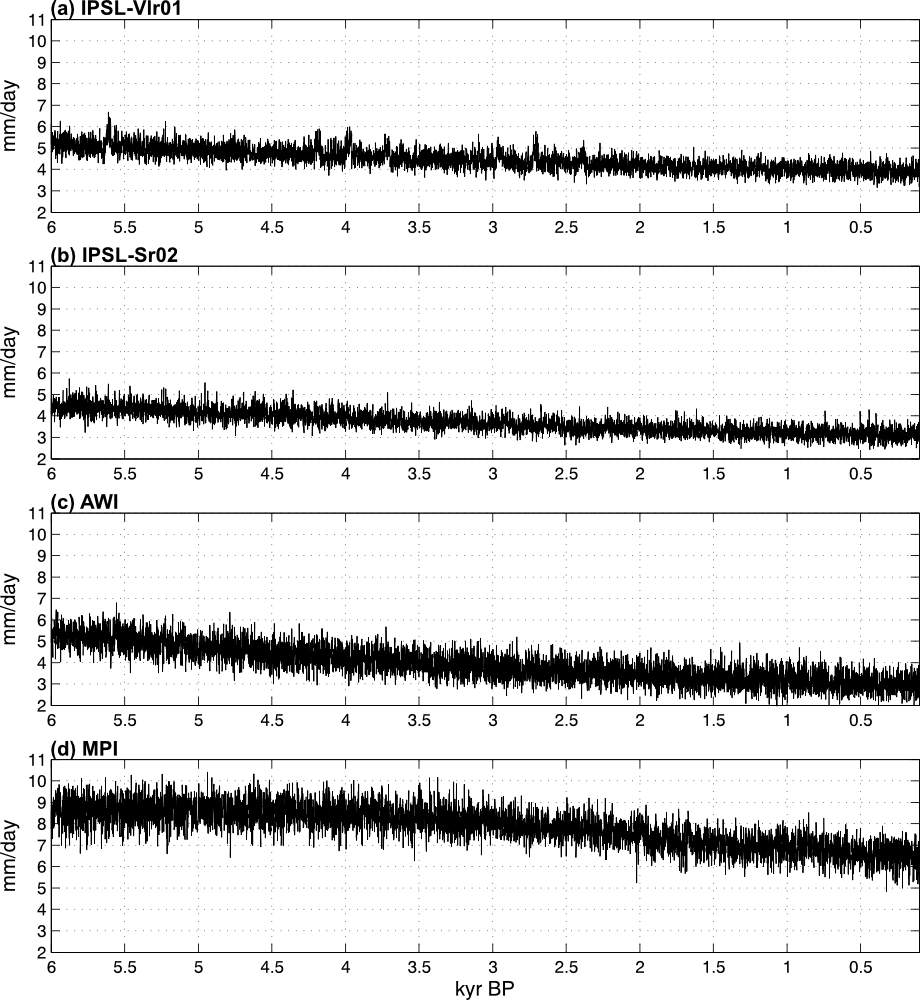


**Figure S2** Mid- to late Holocene evolution of unfiltered WAMR in the (a) IPSL-Vlr01, (b) IPSL-Sr02, (c) AWI and (d) MPI simulations. The WAMR index is defined as the rainfall area-averaged in the 7°-15°N – 15°W-20°E region.

**
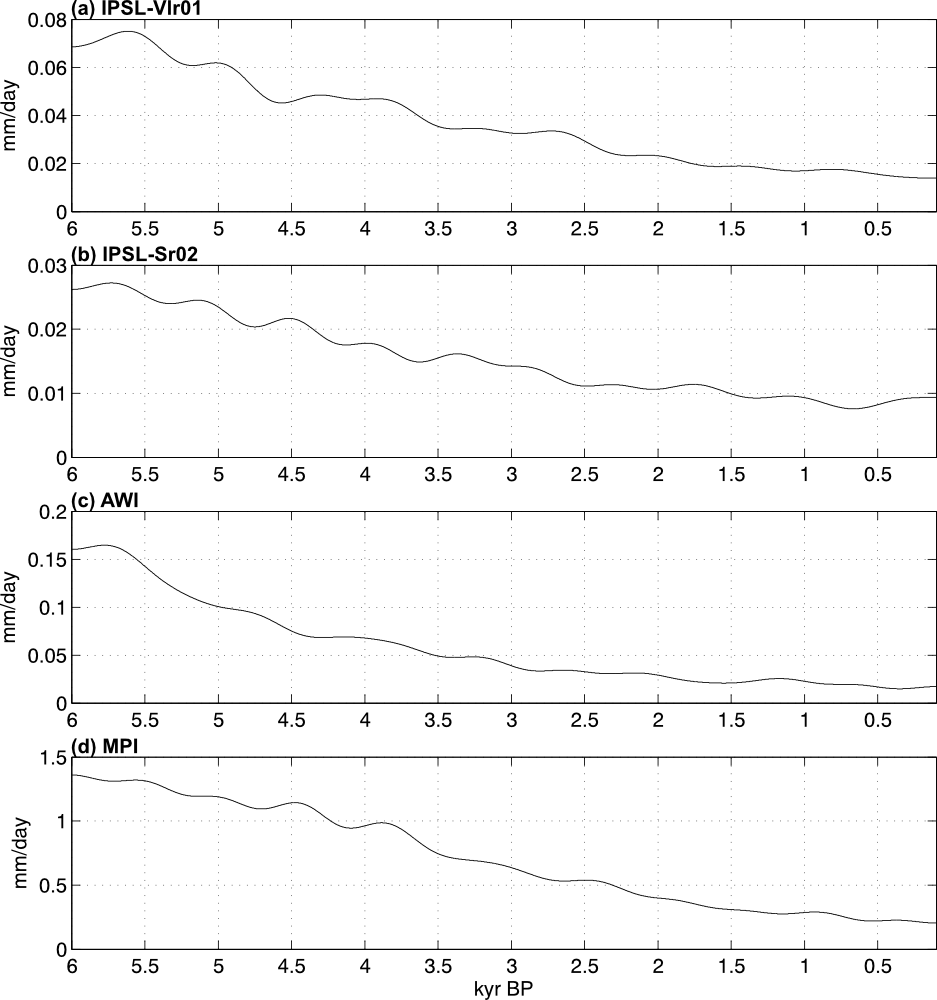
**

**Figure S3** Mid- to late Holocene evolution of the northern edge of 500-year low-pass filtered WAMR in the (a) IPSL-Vlr01, (b) IPSL-Sr02, (c) AWI and (d) MPI simulations. The WAMR index is defined as the rainfall area-averaged in the 20°-30°N – 20°W-5°E region.

**
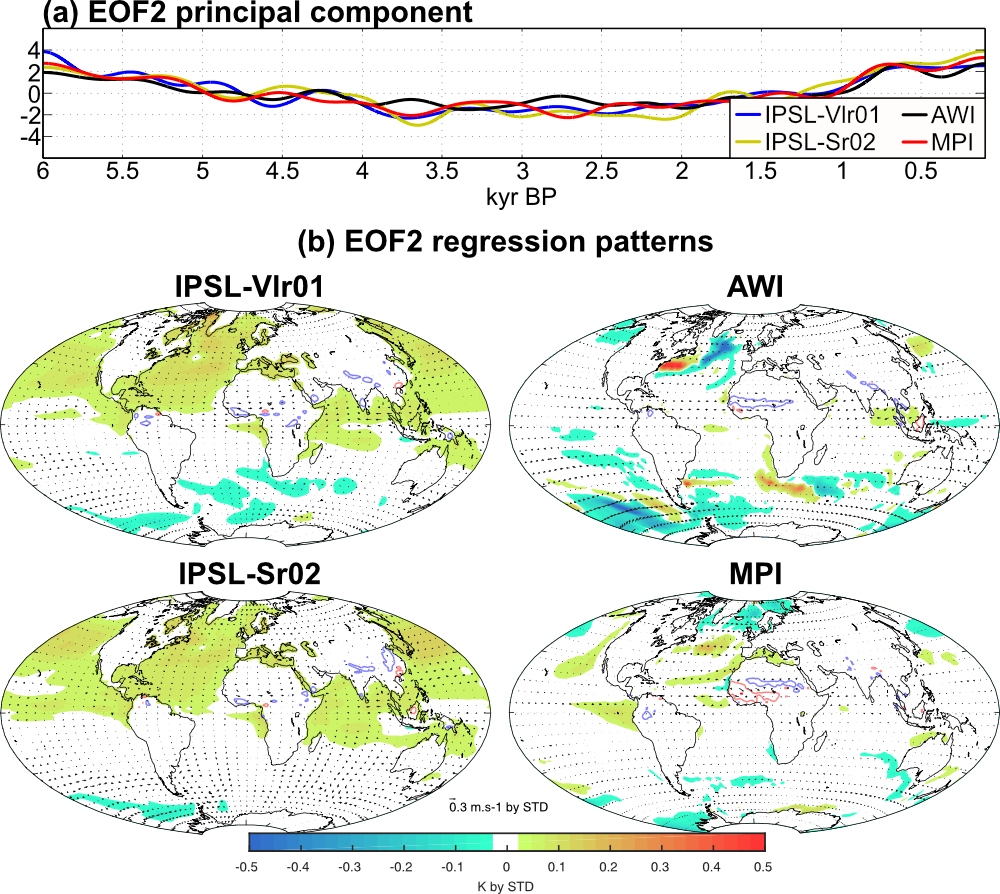
**

**Figure S4** Same as Fig. 5 but for 500-year low-pass filtered anomalies regressed onto the EOF2 principal component.
